# Electrophoretic Profile of Urinary Proteins in Goats During the Peripartum Period

**DOI:** 10.3390/ani16020322

**Published:** 2026-01-21

**Authors:** Berihu Gebrekidan Teklehaymanot, Marilena Bolcato, Gloria Isani, Angelica Lembo, Tolulope Grace Ogundipe, Giulia Ballotta, Francesco Dondi, Arcangelo Gentile, Sabrina Fasoli

**Affiliations:** 1 Department of Veterinary Theriogenology and Welfare, College of Veterinary Science, Mekelle University, Mekelle P.O. Box 231, Ethiopia; berihu.gebrekidan@mu.edu.et; 2 Department of Veterinary Medical Sciences, Alma Mater Studiorum, University of Bologna, Via Tolara di Sopra, 50, 40064 Ozzano dell’Emilia, Bologna, Italy; marilena.bolcato2@unibo.it (M.B.); gloria.isani@unibo.it (G.I.); angelica.lembo4@unibo.it (A.L.); tolulope.ogundipe2@unibo.it (T.G.O.); giulia.ballotta2@unibo.it (G.B.); f.dondi@unibo.it (F.D.); sabrina.fasoli2@unibo.it (S.F.)

**Keywords:** urinalysis, goats, pregnancy, electrophoresis, proteins

## Abstract

Background: Urinary proteins may reflect physiological changes occurring during the periparturient period, but reference data for goats are still lacking. This study investigated urinary protein patterns around parturition to help fill this gap and generate baseline information. Methods: Ten pregnant Alpine goats were sampled by spontaneous voiding 22 ± 3 days before delivery (T0), 7 days postpartum (T7), and 30 days postpartum (T30). Physical and chemical urine analyses were performed, and urinary proteins were separated using one-dimensional sodium dodecyl sulfate–polyacrylamide gel electrophoresis. Statistical tests (Shapiro–Wilk, repeated-measures ANOVA, or Friedman) were applied to evaluate differences among time points. Results: Significant temporal changes were observed: urine pH decreased at T30, the urine protein-to-creatinine ratio increased at T7 and T30, and urinary creatinine concentration was highest at T0. Most samples showed common protein bands at approximately 80, 70, 62, 50, 37, 29, 25, 22, and below 13 kDa, with the 62, 50, and <13 kDa bands present in all samples. Bands between 18 and 64 kDa and above 60 kDa appeared only in some samples. Protein bands between 23 and 42 kDa were more frequent at T0, suggesting immune-related variations associated with pregnancy. Conclusions: This study provides the first description of urinary protein electrophoretic profiles in goats during the periparturient period and highlights measurable changes across time. These findings offer a starting point for developing future research and may contribute to establishing reference parameters for clinical and physiological monitoring in goats.

## Simple Summary

Urine is a valuable diagnostic tool in goats due to its easy, non-invasive collection and stability. Proteins in urine play a key role in many health-related processes. Differences in urinary protein excretion of 10 pregnant Alpine goats before and after delivery were investigated. In detail, urine samples were collected about 22 days before delivery, 7 and 30 days after delivery. All goats remained healthy throughout the study, and urinalysis showed mostly normal findings. Dipstick tests were largely negative, except for frequent protein positivity and occasional red blood cells, ketones, or white blood cells, with no significant differences among the sampling times. Microscopic evaluation confirmed the absence of urinary tract inflammation or infection, while urine pH, specific gravity, creatinine concentration, and the urine protein-to-creatinine ratio showed some time-dependent changes. Electrophoresis revealed similar protein bands across samples, with no significant differences in their distribution among the three collection times. Overall, these findings provide an initial characterization of urinary changes in goats during the peripartum period, supporting future, more detailed investigations.

## 1. Introduction

Goat farming has recently expanded due to its environmental sustainability, climate adaptability, lower methane emissions, and efficient use of diverse vegetation [1,2]. The global shift toward goat farming is particularly evident in regions such as sub-Saharan Africa, where changing climatic conditions and market demands have led to increased investment in goat production systems [3]. Urine has long served as a valuable diagnostic tool in caprine medicine, being used to assess the animals’ metabolic status and to detect conditions such as pregnancy toxemia [4], chronic kidney disease, or to monitor the impact of various dietary interventions [5], and to evaluate the excretion of trace elements [6]. Due to its non-invasive, low-cost, and large-volume collection, along with its relative biochemical stability compared to other body fluids, urine represents an advantageous matrix for clinical investigations [7]. Of the multitude of urinary molecules, proteins are particularly significant due to their involvement in various pathophysiological mechanisms [8].

Proteomics, the study of the complete set of proteins (the proteome) expressed by a cell, tissue, or organism, has gained significant popularity in animal sciences; an increasing number of studies are focused on exploring variations in proteins of tissues and body fluids from various animal species under different conditions, including stress, infectious diseases, and other diseases [9,10,11,12]. However, most researchers studying farm animals have focused on bovine and porcine proteins, leaving ovine and caprine proteomes mostly unexplored [10,13].

This preliminary study investigated the changes in goat urine chemistry and protein electrophoretic profiles before and after parturition, aiming to discover potential biomarkers of renal damage.

## 2. Materials and Methods

### 2.1. Animals and Their Management

This study was carried out on Alpine goat does farmed at the Teaching farm of the Department of Veterinary Medical Sciences of the University of Bologna (Ozzano dell’Emilia (Bologna), Italy) between January and March 2024. In total, 10 does were enrolled in the study, ranging in age from 2 to 9 years.

The does were kept indoors in group housing with straw bedding, and from April to November, they had daytime access to a fenced pasture area of approximately 2500 square meters. During late pregnancy, their diet included 2.0 kg per day of alfalfa hay and 0.3 kg per day of concentrate feed, which contained ingredients such as wheat bran, sunflower seeds, soybeans, barley, corn cobs, dried beet pulp, and a mix of minerals and vitamins. This concentrate had 17.8% crude protein, 3.6% fat, 10.2% crude fiber, and 6.6% crude ash. The concentrate amount was increased to 0.8–1.2 kg per day during lactation. The does had free access to water and salt licks. Natural mating occurred from August to September, with kidding season expected between January and March.

Goats were considered healthy based on their history and clinical signs. Dysuria, pollakiuria, strangury, polyuria, or the presence of blood clots and/or purulent debris in urine or on the vulva were also considered to assess their health status during the study period. Goats were confirmed pregnant by transabdominal ultrasonography between post-breeding days 60 and 70, The main criteria for positive diagnosis of pregnancy were the visualization of a fetus with heartbeat or placentomes.

### 2.2. Urinalysis and Urine Protein to Creatinine Ratio Evaluation

This was a prospective study wherein urine collection was performed by spontaneous voiding early in the afternoon during husbandry practice in sterile cups at three time points. Urines collected in the last 4 weeks of the pregnancy (22 ± 3 days before delivery) were reported as the first time point (T0), the samples collected 7 days after the delivery were reported as the second time point (T7), and the samples collected at 30 days after the delivery were reported as the third time point (T30). Urine samples were visually inspected immediately after collection by the operators. After centrifugation at 1500× *g* for 10 min, the urine specific gravity (USG) was tested by a refractometer (Clinical Protein Refractometer RHC-200, Huake Instrument Co., Ltd., Ningbo, China), and the semiquantitative dipstick test was performed (Mission^®^ Urinalysis Reagent Strips, ACON Laboratories, Inc., San Diego, CA, USA) by putting one drop of urine on each net and comparing by visual inspection the color change in the strip with the graded scales reported by the manufacturer after 60 s. The microscopic examination of urine sediment was performed following the procedure previously reported [14].

Different aliquots of supernatants were stored at −20 °C for subsequent analysis. The urine total proteins (uTP) and urine creatinine (uCrea) were measured using commercial kits (Urinary/CSF Protein, OSR6170, and Creatinine OSR6178, Olympus-Beckman Coulter, Brea, CA, USA) on an automated chemistry analyzer (AU 480, Olympus-Beckman Coulter, Brea, CA, USA). The calibration of both methods was carried out using standard materials, in accordance with the manufacturer’s instructions for urine (Urinary/CSF Protein Calibrator; Urine Calibrator; Beckman Coulter, Brea, CA, USA), and the quality control checks were performed daily using a commercially available control solution (Liquichek, Urine Chemistry Control, Bio-Rad Laboratories, Irvine, CA, USA). The urine protein to creatinine ratio (UPC; mg:mg) was calculated using the following formula UPC = uTP (mg/dL)/uCrea (mg/dL).

### 2.3. One-D-Electrophoresis

Urine proteins were separated using one-dimensional sodium dodecyl sulfate polyacrylamide gel electrophoresis (1D-SDS-PAGE). After thawing and centrifugation at 3000× *g* for 10 min, the urine supernatants were concentrated using spin columns with a molecular mass cut-off of 3 kDa (Vivaspin 500, Sartorius, Göttingen, Germany), following the manufacturer’s instructions.

Fourteen μL of each concentrated sample were loaded onto 4–12% Bis-Tris polyacrylamide gels (NuPage/Thermo Fisher Scientific, Waltham, MA, USA), and electrophoresis was carried out in a vertical mini-protein gel electrophoresis system (Xcell SureLock Mini-Cell, Thermo Fisher Scientific, Waltham, MA, USA) with 2-(N-morpholino) ethane sulfonic acid buffer (MES; NuPage/Thermo Fisher Scientific, Waltham, MA, USA) at pH 7.3 containing sodium dodecyl sulfate (SDS). Each gel was also loaded with standard proteins of known molecular mass (SeeBlue™ Plus2 Pre-stained Protein Standard, Thermo Fisher Scientific, Waltham, MA, USA). The electrophoresis system was connected to a power supply (Power Pack Basic—Bio-Rad, Hercules, CA, USA) with a constant voltage of 200 V for 40 min. The gels were stained with Coomassie staining (Quick Coomassie Stain, Protein Ark, Sheffield, UK). After staining, the gels were digitalized using a densitometer (ChemidocMP, BioRad, Hercules, CA, USA) and the pherograms were obtained using commercial software (ImageLab 5.2.1 version, BioRad, Hercules, CA, USA). One μg of protein, obtained from a solution containing 1 μg/μL of lactate dehydrogenase (LDH), (Sigma-Aldrich/Merck KGaA, Darmstadt, Germany) was added to each sample as an internal standard of quantity.

### 2.4. Statistical Analysis

The Shapiro–Wilk test was performed to assess the normal distribution of the previously displayed data. A *p* > 0.05 was considered indicative of a normal distribution. A comparison was performed among the three time points for dipstick results (i.e., pH, protein concentration, red and white blood cells), USG, uTP, uCrea, UPC, as well as the number of bands detected in the gels after SDS-PAGE with the Repeated measures analysis of variance ANOVA or the Friedman test, depending on whether the distribution was normal or not, respectively. The range of protein molecular mass (MM) (3.6–108.7 kDa) was divided into six classes (1 = 3–23 kDa; 2 = 23–42 kDa; 3 = 42–62 kDa; 4 = 62–82 kDa; 5 = 82–101 kDa; 6 ≥ 101 kDa) and the relative frequency of each class was calculated for each time points. This relative frequency of each class, expressed as a percentage, was calculated by counting the number of bands in each class and dividing the results by the total number of bands. The time points were also investigated as possible sources of differences with the Friedman test. A *p* < 0.05 was considered significant. Statistical analysis was carried out using a commercially available statistical software (MedCalc Software version 19.3.1 MedCalc Software Ltd., Ostend, Belgium; https://www.medcalc.org; accessed on 19 February 2025). Graphics were made using a commercially available statistical software GraphPad (GraphPad Prism version 10.0.0, GraphPad Software, Boston, MA, USA, www.graphpad.com).

## 3. Results

No animals showed signs of disease during the study. The color of the urine was light yellow, and the turbidity ranged from clear to slightly cloudy. Dipstick test indicated negative outcomes for most of the samples at the three time points, except for 27/30 urine samples that tested positive for protein, 1/30 was positive for ketones, 7/30 were positive for red blood cells, and 1/30 was positive for white blood cells. There were no significant differences between the three time points when evaluating dipstick results for ketones, protein, red blood cells/hemoglobin, and white blood cells (*p* > 0.05). The microscopic examination of urine sediment confirmed the absence of urinary tract inflammation and/or infection. Only occasional presence of red blood cells was detected at microscopic urine sediment examination in 7/30 samples. Data on pH, USG, uTP, uCrea, and UPC at each time point are reported in Table 1.

**Figure 1:**
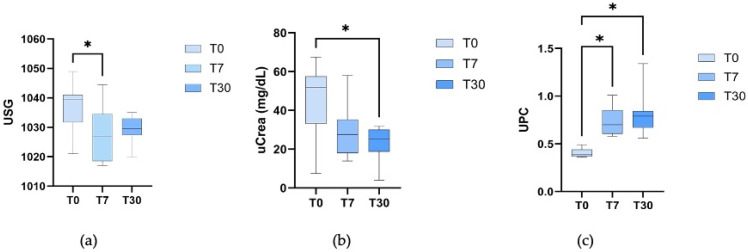
Comparison between three time points: T0 (22 ± 3 days before delivery); T7 (7 days after delivery); T30 (30 days after delivery). (**a**) Comparison of USG; (**b**) Comparison of uCrea concentration; (**c**) Comparison of UPC. The asterisk (*) indicates a statistically significant difference between the time points.

Significant differences in urine pH were observed among the three time points (*p* < 0.00001), with the pH at T30 being lower than at T0 and T7. Additionally, the USG at T7 was statistically lower than that reported at T0 (*p* = 0.03), and the UPC at T7 and T30 showed significant increases compared to the UPC results at T0 (*p* < 0.001) (Figure 1). The uCrea concentration at T0 was statistically higher than at T30 (*p* = 0.0002) (Figure 1). The uTP value did not show a significant difference among time points (*p* = 0.7).

**Figure 2:**
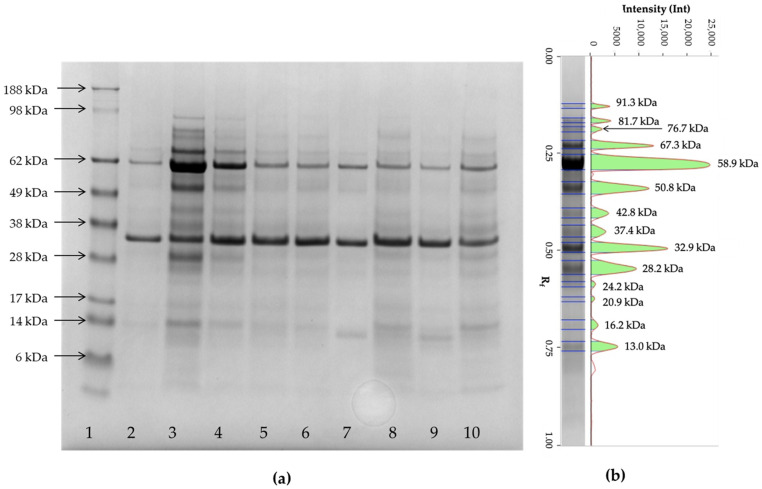
Representative 1D-SDS-PAGE gel and pherograms of urine samples from goats. (**a**) Lane 1 = molecular mass marker (SeeBlue™ Plus2 Pre-stained Protein Standard); lanes 2–8 = urine samples of 3 goats at the three time points. Specifically, from left to right, the order of the samples was T0, T7, and T30 for 3 of the 10 goats included in this study. The molecular masses (MMs) of the marker are reported on the left side of the gel. The band with MMs of 32.9 kDa is the internal standard of quantity (1 μg/μL). (**b**) Representative pherogram of lane 3. The MMs of each band are reported on the right of each peak.

Most of the samples analyzed using 1D-SDS-PAGE exhibited common protein bands with apparent MM of 80, 70, 62, 50, 37, 29, 25, 22, and lower than 13 kDa (Figure 2). The band with the MM at 62 kDa was present in all of the samples analyzed, as well as the bands at 50 and lower than 13 kDa. Conversely, bands with MM between 18 and 64 kDa and those with MM higher than 60 kDa only appeared in some samples.

To compare the protein profiles of the different samples, the electropherograms were divided into six zones based on the molecular mass of the proteins (MM), ranging from small (3–23 kDa) to large (>101 kDa). There was no significant difference in the frequency of the protein bands in the three time points. The data on the frequency of each class of bands at each time point are reported in Table 2.

## 4. Discussion

This study evaluated changes in urinary protein and protein profiles in goats before and after parturition, specifically at 7 and 30 days postpartum. Proteinuria was present in these animals, albeit with a limited magnitude. The median UPC value reported in this study was higher at T7 and T30 than at T0. According to the data reported, the overall median value at T0 is also higher than the reference interval for healthy ruminants (UPC > 0.25) [15].

Positive results were found for some analytes at the dipstick test. The positivity to ketones was found to be 1+ (10 mg/dL) only in one specimen at T7. Despite its inclusion might be questioned, this goat did not show clinical signs during the entire study period, referring to causes of ketonuria, such as the pregnancy toxemia and fatty liver syndrome [16]. Moreover, except for a slight effect on USG values, the presence of ketones in the urine had no effect on the other variables, and their concentration is considered abnormally elevated when about 100 mg/dL [15,17]. Additionally, this result could be explained by considering the metabolic switch of glucose metabolism for lactose synthesis and milk production or by deriving from rumen ketone bodies [8,15]. The positivity of proteins was irrespective of the time points studied, and it cannot be excluded that these results might be a false positive, as an alkaline pH could be responsible for up to 2+ protein positivity in goats, and high false positive protein reactions in cattle [17,18]. Moreover, a weak correlation was reported in cows between dipstick protein test and UPC values, highlighting that these data should be cautiously interpreted and always in conjunction with pH and UPC ratio to exclude proteinuria [15,18]. Additionally, all animals at each time point exhibited USG values exceeding 1.020–1.025, which is regarded as indicative of adequate concentrating ability in ruminants [17,19], further supporting that the kidneys were properly functioning as the goats enrolled in this study remained well-hydrated and did not show clinical signs referring to renal impairment. Although the majority of the dipstick results and USG did not show differences between time points, nearly all of the urinalysis results observed in this study, across the three time points, align with those reported for sheep, goats, and cows [15,17].

The UPC values at T7 and T30 were significantly higher than those reported during pregnancy (T0); however, their median values exceeded the reference intervals reported for healthy ruminants (UPC > 0.25) at all time points [15]. Since a UPC ratio range of 0.21–1.34 was reported in healthy sheep, according to the upper limit of the reference interval, the occurrence of pathological proteinuria would be defined only at T30 [20]. However, Athanasiou et al. [20] highlighted the presence of glomerulonephritis in these animals, leading the authors to select a UPC ratio of 0.2 as a cutoff for proteinuria in sheep as well. To the best of the authors’ knowledge, no reference intervals have been reported for UPC in goats, and this paucity of data might limit the interpretation of our results. According to Hermann et al. [15] and Athanasiou et al. [20], proteinuria was already present at T0 and continued to increase over time, with no significant difference observed between T7 and T30. Similar data are reported for women during normal pregnancy, as a rise in protein excretion can also persist in the postpartum period, taking 5 to 6 months to resolve [21]. The reasons behind this change in protein excretion might be due to increased glomerular filtration rate (GFR) coupled with increased permeability of the glomerular basement membrane, and/or increased tubular proteinuria [22]. The prevalence of twin pregnancies in our study group (3 goats: singletons; 7 goats: twin pregnancies) could further influence the amount of protein excreted in urine, as twin pregnancy is known to cause greater protein excretion than in singletons in humans and affect urinary electrolyte excretion in White Improved goats [23,24].

Another possible explanation for the rise in UPC values over time could be the concurrent decrease in urinary creatinine concentration (i.e., uCrea at T0 is significantly higher than at T30), while uTP values remained almost constant during the study period (no differences among groups detected). Consequently, the increase in UPC at T7 and T30 might result from a reduction in urinary creatinine concentration rather than a worsening of proteinuria, as reported in cattle, wherein a decrease in urinary creatinine concentration in the postpartum period was identified between 13 and 60 days after parturition [25]. However, data are lacking, and further studies are required to investigate a similar scenario also in peripartum goats.

An additional explanation for the decrease in uCrea at subsequent timepoints in our population may be related to the decrease in the USG during the study period, despite it not being significant in the post-partum period. In fact, a reduction in urine concentration is also associated with a reduction in uCrea concentration. A reduction in the USG, however, is not strictly related to the occurrence of a renal pathological process but might be the result of increased water intake. Nevertheless, the USG values at all time points were indicative of an adequate capability of the kidney to concentrate urine.

Collectively, our findings suggest that renal damage is unlikely in these animals at any time point. Additionally, they align with previous data reported for pregnant cattle, where the UPC value is lower during pregnancy than in the postpartum period [8]. This scenario is more likely due to pregnancy-related physiological changes rather than pathological findings. However, given the paucity of data in small ruminants, further studies are needed to corroborate these findings and establishing UPC reference intervals in goats would be beneficial to enhance understanding of protein excretion during the peripartum period in this species.

To date, few studies have used 1D-SDS-PAGE electrophoresis to analyze urine samples in goats. In this species, little has been published on the proteins expressed during pregnancy in various biological samples, such as blood, placenta, or urine, using different methodologies, including 1D-SDS-PAGE [26,27,28,29,30,31]. In the absence of reference goat urinary proteome, the identity of some of the bands can be hypothesized based on their apparent MM and by comparing the profiles with those previously reported in humans and other mammals. The most intense protein bands observed in this study correspond to approximate molecular mass of ~70, ~62, ~55, and ~25 kDa. These bands may tentatively align with proteins such as transferrin, albumin, and the heavy and light chains of immunoglobulins; however, in the absence of confirmatory analyses (e.g., LC–MS/MS or immunoblotting), these assignments should be regarded as preliminary and purely indicative rather than definitive protein identification. These proteins have been identified in the urine of different species of mammals, including dogs, cats, goats, camels, cows, giraffes, and California sea lions [14,32,33,34,35,36,37,38,39,40,41,42,43,44]. Transferrin, which appears as a very thin band in the majority of the urine analyzed, is the iron transport protein in plasma and a negative acute phase protein (APP), with its major diagnostic application being related to its role in diseases of iron metabolism [45]. The increase in transferrin concentration in goat serum has been recently proposed as a potential biosensor for stress [39,46]. Albumin is the most abundant protein found in serum. It is physiologically present in trace amounts or in low concentrations in the urine of various species of healthy animals or humans [14,33,37,39,40,42,44,45,47,48]. Fluctuations of this protein in serum have been observed in response to elevated stress levels and in response to environmental enrichment in dry and milking goats [46]. However, as reported for women, an increase in protein excretion might be expected in the postpartum period coupled with increased permeability of the glomerular basement membrane, or increased tubular proteinuria [21,22]. A similar scenario is also possible in goats, and qualitative and quantitative changes in protein excretion are expected over time.

For diagnosing pregnancy, two other proteins have been investigated in goats: pregnancy-specific protein B (PSPB) and pregnancy-associated glycoproteins (PAGs), as they are highly specific for pregnancy [49]. The PSPB was isolated from caprine plasma and ovine placenta, and its concentration in pregnant goats was significantly higher by 24 days after breeding compared to non-pregnant animals, as well as in French Alpine goats with two fetuses compared to specimens with one embryo [26,49]. The MM of variants isolated from the ovine placenta ranges from 43 kDa to 66 kDa, similar to the protein purified from bovine serum (MM of 67 kDa), moose (MM of 31–58) and elk placenta (MM of 31–57 kDa) [38,50]. This protein is detectable in the bovine maternal blood until 87–105 days post-partum, although the decrease in its concentration begins before calving and might correlate with the decline in the binucleate cell count [51].

PAGs have been reported in goats primarily in serum or placental tissue, with an MM ranging from 42 to 62 kDa [27,29,30,31]. The concentration of these proteins increased starting from 1-week post-artificial insemination and rising 10-fold times after 2.5 months of mating, compared to non-pregnant goats [29,31]. Moreover, the concentrations significantly differed between pregnant and non-pregnant goats by day 21. After parturition, they decreased rapidly, returning to lower levels after four weeks postpartum [49,52]. As their concentration fluctuates significantly during the peripartum period and remains high for weeks after parturition, this may also explain the varying intensity of the bands in our studied animals across the three time points. However, to date, these proteins have not been identified in urine, and further studies are needed to confirm this hypothesis to understand how these proteins change before and after pregnancy in goats.

While the difference was not statistically significant, goat urine exhibited a higher relative frequency of protein bands in the 23–42 kDa range at T0 and T7 than at T30. Different proteins have MMs in this range, including haptoglobin (MM of 37 kDa) and cathelicidin (MM ranging from 14 to 25 kDa). In cows, haptoglobin and cathelicidin increase during the early days of gestation [43]. Haptoglobin is an acute-phase glycoprotein, synthesized in the liver where it is cleaved into a light α chain and a heavy β chain, with a different MM ranging from 9 to 40 kDa [53]. A significant increase in its concentration can be detected after three weeks of gestation in dogs [54]. Cathelicidins are host defense peptides (HDPs), which have antimicrobial and immunomodulatory functions [55]. However, these proteins have not yet been identified in the urine of goats, and further studies are required to confirm their presence. Other proteins to take into consideration are heavy and light chains of immunoglobulins (MW of 55 and 25 kDa, respectively), lysozyme and β2-microglobulin (MM of <13 kDa), similar to the pattern of urinary protein previously reported in cows and heifers by Ferlizza et al. [8]. Lysozyme, which is involved in pathways related to neutrophil degranulation, functions as an antibacterial enzyme and has also been proposed as a suitable biosensor for stress in goats’ peripheral blood [44,46,56].

Notably, the majority of the proteins discussed in this study are involved in immune activities, and pregnancy is viewed as a pro-inflammatory state that necessitates physiological adaptation of the mother’s immune system to guarantee the protection of the fetus and its tolerance from her body [54]. Additionally, mild to moderate inflammation and the resulting degenerative changes, caused by cytokine and APPs release, have been identified as potentially halting fertilization through hormonal imbalances and leading to temporary infertility in infected does [57]. Given this scenario, it is conceivable that complex adaptations occur in the peripartum period in goats to ensure the survival of the fetus; therefore, further study is required to deepen the understanding of protein changes in this species during pregnancy.

This study has some limitations to note. First, the small sample size used in this study may limit the power of the results; however, it provided a first insight into the peripartum change in urinary protein excretion in goats, which requires further investigation. Moreover, the absence of a UPC reference interval in goats could have limited data interpretation, as a baseline for non-pregnant animals is lacking. Although the ruminants might share common renal physiology features, each species-specific peculiarity should be assessed. Therefore, further studies are needed to confirm our findings and determine if proteinuria also occurs in pregnant goats as a physiological change. However, our data are consistent with those reported in other ruminants and women, suggesting that both the small sample size and the absence of a UPC reference interval may not have substantially affected the results. Another limitation is the discrepancy between the protein quantification by the pyrogallol red-molybdate method (to assess UPC value) and SDS-PAGE electrophoresis, which requires further studies to quantify the magnitude of this discrepancy. Finally, protein identification by mass spectrometry is needed to confirm the presence of the proteins just hypothesized in caprine urine based on their apparent MM.

## 5. Conclusions

Our study examined how urinary protein excretion changes during the transition from late pregnancy to the postpartum period in goats, revealing physiological adaptations associated with this critical phase. Although validation with a larger population and inclusion of qualitative protein identification using mass spectrometry are needed, the present findings provide new insights into the dynamics of the caprine urinary proteome. Notably, some adaptive mechanisms observed in goats suggest potential scientific value and open avenues for future research.

## Figures and Tables

**Table 1:** Data on pH, USG, uTP, uCr, and UPC are presented for each time point. Data are reported as the mean ± SD or the median (min–max), depending on whether the data are normally distributed or not.

Variables	N	T0	T7	T30
pH	10	9 (9–9)	9 (9–9)	8.5 (8.5–9)
Dipstick protein (mg/dL)	10	100 (30–500)	100 (0–500)	100 (0–500)
USG	10	1.037 ± 8	1.028 ± 9	1.030 ± 4
uTP (mg/dL)	10	21.10 ± 4.90	20.50 ± 8.34	20.50 ± 7.98
uCrea (mg/dL)	10	46.47 ± 18.12	28.54 ± 13.19	23.07 ± 8.87
UPC	10	0.39 (0.36–0.49)	0.70 (0.58–1.01)	0.79 (0.56–1.34)

T0 (22 ± 3 days before delivery); T7 (7 days after delivery); T30 (30 days after delivery).

**Table 2:** The band number and the relative frequency of the molecular mass (MM) classes for each time point are reported. Data are reported as median (min–max). N indicates the number of specimens included in each group.

		Frequency		
	N	T0	T7	T30
Band number	10	4.5 (2–10)	4 (1–13)	6 (2–12)
MM class 1 (3–23 kDa)	10	0 (0–27.3)	9 (0–25)	0 (0–22.2)
MM class 2 (23–42 kDa)	10	22.5 (0–33.3)	21.55 (0–33.3)	8.35 (0–50)
MM class 3 (42–62 kDa)	10	38.10 (0–66.7)	36.65 (0–100)	33.3 (22.2–66.7)
MM class 4 (62–82 kDa)	10	29.2 (0–50)	20 (0–50)	21.6 (11.1–50)
MM class 5 (82–101 kDa)	10	0 (0–20)	0 (0–9.1)	0 (0–16.7)
MM class 6 (>101 kDa)	10	0 (0–0)	0 (0–0)	0 (0–7.7)

T0 (22 ± 3 days before delivery); T7 (7 days after delivery); T30 (30 days after delivery).

## Data Availability

All study data are contained within the article.

## Abbreviations

The following abbreviations are used in this manuscript:

1D-SDS-PAGE	One-dimensional sodium dodecyl sulfate polyacrylamide gel electrophoresis
APP/s	Acute phase protein/s
HDPs	Host defense peptides
MM	Molecular Mass
PAGs	Pregnancy associated glycoproteins
PSPB	Pregnancy-specific protein B
RBP	Retinol-binding protein
uCrea	Urine total creatinine concentration
UPC	Urine protein–creatinine ratio
USG	Urine specific gravity
uTP	Urine total protein concentration
